# BI-SENT: bilingual aspect-based sentiment analysis of COVID-19 Tweets in Urdu language

**DOI:** 10.1371/journal.pone.0317562

**Published:** 2025-06-13

**Authors:** Ehtesham Hashmi, Amna Altaf, Muhammad Waqas Anwar, Muhammad Hasan Jamal, Usama Ijaz Bajwa

**Affiliations:** 1 Department of Information Security and Communication Technology, Norwegian University of Science and Technology, Innlandet, Norway; 2 Department of Computer Science, COMSATS University Islamabad, Lahore, Pakistan; 3 Department of Computer Science, Government College University Lahore, Lahore, Pakistan; Government College University Faisalabad, PAKISTAN

## Abstract

The COVID-19 pandemic resulted in over 600 million cases worldwide, and significantly impacted both physical and mental health, fostering widespread anxiety and fear. Consequently, the extensive use of online social networks to express emotions made sentiment analysis a crucial tool for understanding public sentiment. Traditionally, sentiment analysis in the Urdu language has focused on sentence-level analysis. However, aspect-level sentiment analysis is increasingly important and remains underexplored due to the challenges of the costly and time-consuming manual dataset annotation process. This study presents an innovative bilingual aspect-based sentiment analysis for Urdu and Roman Urdu using unsupervised methods. For Urdu, a syntactic rule-based approach achieves an accuracy of 83% in extracting aspect terms, marking a 5% improvement in F1-score over existing methods. For Roman Urdu, the study employs collocation patterns and topic modeling to identify and categorize key aspects, resulting in a perplexity score of –7 and a coherence score of 41. The results not only demonstrate the semantic coherence of the identified categories but also represent a significant advancement in aspect-level sentiment analysis by eliminating the need for manual annotation. This study offers new insights into the sentiments expressed during the pandemic, providing valuable feedback for policymakers and health organizations.

## Introduction

The coronavirus (COVID-19) was first reported in Wuhan, China, in 2019 and spread rapidly globally [1]. Governments took drastic measures and hand sanitization, face masks, and social distancing were enforced to prevent the spread and impact of the disease [2]. Large gatherings were outlawed, educational institutions were closed, and international traveling was suspended [3]. Consequently, along with underdeveloped and developing countries, developed countries were also relentlessly affected by the pandemic. As the pandemic influenced the normal course of life and captivated the world with huge intensity, individuals around the globe took to Online Social Networks (OSNs), that is the primary source of information for most people nowadays, to share their views and emotions regarding the pandemic [4]. Social media sites, such as X (formerly Twitter), showed a huge spike in the number of tweets on novel coronavirus in a very short duration. To gain valuable insights into emotions, public attitudes, and reactions toward different aspects of the pandemic including social issues, healthcare measures, government policies, and personal experiences, Sentiment Analysis (SA) has been widely used by researchers by utilizing the data from OSNs like X (formerly Twitter) [5–10].

SA is mostly performed at three levels i.e., document level, sentence level, and aspect level [11]. Document-level SA assigns the polarity (positive, negative, and neutral) to the overall document, and sentence-level sentiment classifies the sentence as positive negative, and neutral. Furthermore, it provides a coarse-grained analysis as compared to document-level SA. Aspect level SA is the most fine-grained analysis as it identifies the sentiment as well as the aspect being discussed in the sentence. Additionally, it identifies four types of information from data i.e., extraction of Aspect Term/s (ATE), Aspect Term/s Polarity (ATP), Aspect Category (ACT), and Aspect Category Polarity (ACP). ATE is the actual word or phrase that appear in a text indicating an aspect category. ATP is the sentiments (positive, negative, neutral) associated with these aspects [12]. ACT represents a unique aspect of an entity. The main difference between ATE and ACT is that ATEs are more fine-grained and appear in user reviews while ACT is the coarser category of the sentence and does not appear in the sentence. Categories are inferred based on adjectives, or the context of the sentence rather than being extracted from aspects. ACP is the sentiment associated with the category [13].

Different approaches are used to perform SA at different levels including lexicon-based approach, Machine Learning (ML) and Deep Learning (DL) based approach, and hybrid approach. The lexicon-based approach relies on lexicons and rules-based methods for sentiment classification. ML/DL methods work by training the ML/DL models on training data and the trained models are then used for predictions. The hybrid approach mostly uses ML/DL methods combined with the lexicon-based approach for sentiment classification [14]. Existing studies on Urdu/Roman languages are mostly at the sentence level, and only a few studies exist in aspect-based sentiment analysis (ABSA) which is the most fine-grained analysis. Furthermore, existing studies on ABSA mostly rely on supervised ML approaches which require labeled data for the classification of aspects and sentiments associated with them. Manually labeling a dataset is a laborious, time-consuming, and costly process. Therefore, this study presents Bilingual Aspect Based Sentiment Analysis (BI-SENT) using unsupervised approaches i.e., syntactic rule-based approach, and topic modeling methods. We consider Urdu and Roman Urdu languages. Urdu is a resource-scarce Asian Language having 100 million speakers all around the globe. It is derived from various languages including Persian, Arabic, English, Sanskrit, Turkish, and Hindi. It is a morphologically rich language having phrases and constituent words that tend to be more complex because of variations and recurrent derivations. Urdu is written from right to left using Arabic script and Roman Urdu is written from left to right using Latin script [15]. Due to the difference in both languages as to how these languages are written, we have to handle them separately rather than considering the text as code-mixed. For this purpose, we have developed a parallel corpus on COVID-19 for both languages. For the Urdu language, a rule-based approach is used to extract the aspect terms, aspect term polarity, and aspect categories while for Roman Urdu, collocations are used to extract aspect term/s, and topic modeling is used to extract aspect categories. The main contribution of this study is summarized as follows:

- We develop a benchmark parallel dataset in Urdu and Roman Urdu ABSA on COVID-19 tweets.
- We extract three types of information in our dataset for Urdu i.e., ATEs, ATP, ACT, and two types of information from Roman Urdu i.e., ATEs and ACT using an unsupervised approach.
- We propose six syntactic rules for the classification of ATEs, ATP, and ACT in Urdu. To the best of our knowledge, we made a pioneering effort to solve the problem of ABSA in Roman Urdu by leveraging collocation patterns and topic modeling.

The remainder of the paper is organized as follows. The Related Work section presents the literature review on the SA of Urdu and Roman Urdu languages. The Methodology section discusses the research methodology. The next two sections discuss the experimental setup and results and discussion followed by the Conclusion section which summarizes the findings of this paper.

## Related work

SA focuses on determining the sentiments expressed in the text and has recently gotten the attention of researchers due to its applications in various domains. It is widely performed using different approaches i.e., lexicon-based, ML/DL approaches, and hybrid approaches. This section presents a comprehensive literature survey on SA at various levels.

### Urdu language

Altaf et al. [15] perform SA at the aspect level following the standards of SemEval-2015 in the Urdu language. Four types of information are identified during the annotation process including aspect, aspect category, aspect polarity, and aspect category polarity.TF-IDF and n-grams are used for feature extraction and K Nearest Neighbor (KNN), Random Forest (RF), and NaÃ¯ve Bayes (NB) are used for feature classification.

Sadia et al. [16] elaborated a lexicon and rule-based approach for SA in the Urdu language focusing on three main approaches. The first method is supervised, the second is lexicon rule-based, and the third is a hybrid method. Lexicon-based methods do not yield high accuracy as they heavily depend on the size and quality of training data and because many lexical objects have a positive script in one domain while a negative script in another domain. Sentiment-annotated lexicons are produced that add subjectivity-related details to a word’s syntactic and morphological characteristics. Using the shallow parsing technique, Syed et al. [17] perform ABSA for the Urdu language. They identify and extract the SentiUnits which contain the aspects having some sentiment information. For this, a lexicon-based approach is employed. The dataset comprises instances of product and movie reviews. They achieved an accuracy score of 0.72 and 0.78 respectively.

Mukhtar et al. [18] use a rule-based approach and unlike other approaches that mostly focus on nouns, adjectives, and negations, they also focus on verbs and other context associating words. The dataset was collected from online Urdu blogs related to the 14 different genres. Due to the addition of more rules and the expansion of the sentiment lexicon with more words, their approach achieves higher precision and accuracy scores. To perform ABSA in the Urdu language, Rana et al. [19] adopt a rule-based methodology and manually create various syntactic rules for identifying opinion words and their targets using this technique. To identify opinion words in a sentence and related targeted words/phrases, these rules make use of opinion lexicons. Polarities are assigned to each aspect after extracting the associated aspects. The identifier performs more effectively with the rules than without them. Ahmad et al. [20] propose an approach for ABSA in the Urdu language following SemEval-2014 guidelines. Hand annotations are performed for the aspect terms, aspect categories, and aspect polarity.

Hassan et al. [21] use Bag of Words (BOW) model and SEGMODEL to perform SA in the Urdu language on a dataset comprising reviews of 401 electronic devices and reviews of 443 automotive parts. The BOW model is used in their first experiment, and SEGMODEL is used in the second independent experiment. The identification of sub-opinions is the primary goal of this study. Most studies focus on one opinion while this study handles multiple opinions, also known as sub-opinions. Ahmed et al. [22] propose a new Meta-Learning Ensemble (MLE) method for the Urdu language SA by incorporating both ML and DL algorithms. DL models need a large dataset to perform well while meta-learning needs only a few tasks to give the expected output. The proposed ensemble technique combines the predictions of both the inter- and intra-committee classifiers on two separate levels, resulting in reduced training complexity and overfitting. The dataset comprises 28,921 Urdu language reviews with positive, negative, or neutral labels. N-gram along with other word embedding including Continuous Bag of Words (CBOW) and skip-gram are used. The proposed MLE method outperforms the baseline achieving an accuracy of 86.42%.

Altaf et al. [23] perform SA at aspect level in Urdu language. Sports tweets are collected from X (formerly known as twitter) and fine-grained SA is performed by extracting ATEs ATPs, ACTs and, ACPs using ML and deep learning approaches. Additionally, implicit ATEs are also identified from the data. For the task of ATPs, ACTs, and ACPs classical ML outperformed DL models while for ATEs DL approach performed best as compared to traditional ML classifiers.

Shabbir et al. [24] purpose a framework for Urdu SA using DL techniques which comprises of data collection, preprocessing, and sentiment classification modules. A publicly available movie dataset i.e., IMBD is translated in Urdu and three deep learning models i.e., 1-Dimensional Convolutional Neural Network (1D-CNN), Multilingual-MiniLM-L12-H384 transformer, and Long Short-Term Memory (LSTM) are used for SA. The experimental results illustrate that Multilingual-MiniLM-L12-H384 transformer based model is most suitable architecture for SA that acheived the highest accuracy of 89%.

Ashraf et al. [25] use deep learning approach for Urdu Text Sentiment Analysis (USA-BERT) using Bidirectional Encoder Representations. An Urdu dataset for SA-23 (UDSA-23) is introduced for the classification of sentiments. Their proposed approach comprises four modules i.e., preprocessing of data using BERT tokenizer, creation of BERT embeddings for each review, and fine tuning of deep learning BERT classifier. In the last module, Pareto principle is employed on two state-of-the-art datasets (UDSA-23) and (UCSA-21) to evaluate the performance of USA-BERT. The experimental results show that USA-BERT outperformed existing techniques by significantly improving the accuracy by 26 and 25% respectively.

Altaf et al. [26] perform SA on Urdu language sentence level using news dataset. A dataset is developed that comprises idioms, proverbs, and news domain. Different types of linguistic features are extracted from the data which includes part-of-speech tag-based features, numeric features, and boolean features. Then, machine learning approach is used for the classification of sentiments and several machine learning classifiers are used. J48 performed best with the accuracy of 90% and F-measure of 88%.

### Roman Urdu

Zahid et al. [27] introduce the task of Aspect Based Opinion Mining (ABOM) for Roman Urdu which involves extracting an aspect of the sentence after classification and giving that aspect a polarity. Different Supervised ML approaches are used, including SVM, Decision Tree (DT), LR, RF, and KNN. The reviews are classified into the relevant category using the Support Vector Classifier (SVC). Rana et al. [28] propose an unsupervised approach to SA for Roman Urdu. First, the proposed model normalizes the text to account for different word spelling variations. They use Roman Urdu and English opinion lexicons after normalizing the text to identify users’ opinions from the text correctly. Negation terms and stemming are used to assign polarities to each extracted opinion. Additionally, the proposed model assigns a score to each sentence based on the polarities of extracted opinions and categorizes each sentence as positive, negative, or neutral. They validate their approach using two publicly available datasets for Roman Urdu and compare it with an existing model.

Mahmood et al. [29] perform SA for Roman Urdu using rule-based, N-grams, and Recurrent Convolutional Neural Network (RCNN). They perform binary and tertiary classification and yield an accuracy score of 65% and 57% for binary and tertiary classification problems, respectively. Ali et al. [30] perform ABSA in Roman Urdu/Hindi and English language on Uber reviews collected from Facebook. The reviews are converted into English using APIs and multiple aspects are extracted using Parts of Speech (POS) features like nouns, adverbs, and adjectives to get prominent aspects, and polarity is assigned to every aspect. Lexicon-based, ML and DL approaches are used to get better results. Khan et al. [31] use the CNN-LSTM-based approach for the Roman Urdu. They also use the SVM classifier and Word2Vec CBOW word embedding models that prove to be more beneficial for Roman Urdu achieving 90% accuracy. They also use TF-IDF and GloVe word embeddings and RF classifier that achieves an accuracy of 77% for the RUSA (Roman Urdu Sentimment Analysis) dataset. Nagra et al. [32] perform deep SA for Roman Urdu using the Faster Recurrent Convolutional Neural Network (FRCNN) model on the RUSA-19 dataset. They also use RNN, rule-based, and N-grams models for better results Although RNN takes less time than RCNN, RCNN performs better than the baseline. The proposed RNN and RCNN models achieve 91% and 92% accuracy respectively, outperforming the baseline. Chandio et al. [33] propose a deep recurrent architecture for Roman Urdu using attention-based RU Bidirectional LSTM (RU-BiLSTM). For the complete coverage, they focus on both direction and context-based analysis. Word2Vec, GloVe, and FastText word embedding techniques are used. RUSA-19 and RUECD (Roman Urdu e-commerce) datasets are used which outperform the baseline achieving promising results. Using Word2Vec embedding with BiLSTM achieves an accuracy of 87%.

Li et al. [34] propose a CNN-based approach with transfer learning and attention mechanism to improve the performance of SA for Roman Urdu and Hindi languages. Various word embedding techniques are used including Word2Vec, TF-IDF, GloVe, and FasText. To reduce the number of dimensions in the dataset, an average pooling mechanism is implemented along the hidden layers, achieving an accuracy of 80% with a supervised algorithm for 15,000 user reviews and 92% accuracy with the DL algorithms. Qureshi et al. [35] perform SA of Roman Urdu language using reviews of Pakistan Super League’s (PSL) official song collected from X (formerly Twitter) and Facebook. Five main algorithms including four ML algorithms: NB, RF, LR, KNN, and artificial neural network are used. Each review is either tagged positive or negative. Using NB and LR an accuracy of 97% is achieved. Kabra et al. [36] use CNN-based model and TF-IDF vectorization for word embedding. They stacked different ML models with CNN including SVM, Word2Vec, and LSTM, and CNN+LSTM achieved the highest accuracy of 86.03%. Manzoor et al. [37] use self-attention BiLSTM for the sentence level SA in Roman Urdu that extracts fine-grained information from the sentences. The text representation is done using LSTM. They perform binary classification on a dataset comprising 10,000 instances and achieve 68.4% accuracy using BiLSTM.

## Methodology

**Fig 1:**
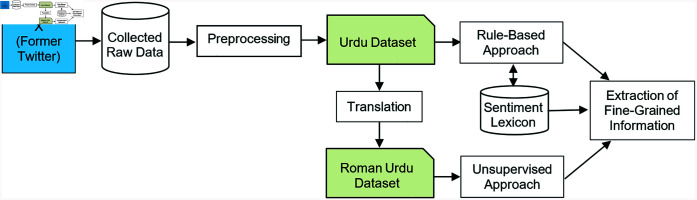
Flow of bilingual aspect based sentiment analysis.

This section explains the methodology of the proposed BI-SENT adapted to perform ABSA in Urdu, as shown in Fig 1. In our SA framework, we utilize a Rule-Based approach, complemented by a Sentiment Lexicon, to analyze Urdu text effectively. This approach involves syntactic rules that have been custom-designed to identify and classify sentiment expressions accurately. The Sentiment Lexicon supports this by providing precise polarity assessments for terms identified by the rules, ensuring accurate sentiment classification. This combination allows for a thorough analysis, capturing both explicit expressions and subtle linguistic cues in the Urdu language. Urdu is written from right to left in the Nastaleeq style that includes Arabic character sets comprising diacritical marks, basic and secondary letters, special symbols, and punctuations [38]. It is a morphologically rich language and multiple variants of a single word exist making it more challenging to deal with. Roman Urdu is written from left to right using Romanagari script that uses English alphabets making it easier to type using English keyboards, and is widely used by billions of people from the Indian Subcontinent and worldwide [39].

Roman Urdu faces several challenges: the absence of standard spelling conventions leads to inconsistent and varied written communication; the phonetic sounds unique to Urdu are often lost in translation when using Latin script, diminishing its rich literary nuances; the language’s complexity is evident in its poetry and multiple meanings often ascribed to single words, and dealing with multiple sentences having the same meaning or a single word refereeing to two or more similar sounding words [40], i.e., “Umer nay phool tora” and “Aik phool Umer nay tora” both means “Ali Plucked a flower” and “Khawer” means “Dawn” as well as “miserable” depending on how it is pronounced. Due to the difference in nature of both languages, we deal with them separately. To classify the ATEs, ATP, and ATC for the Urdu language, we use a syntactic rule-based approach by proposing six rules, and for Roman Urdu, we employ an unsupervised approach since there is no POS tagger available and collocations are used to classify ATEs, and topic modeling is performed to classify ATC.

### Dataset collection

Due to the unavailability of publicly available datasets for ABSA in the Urdu language, for this study, we develop a new dataset comprising tweets related to COVID-19. Initially, a total of 7,230 tweets were extracted from X (formerly Twitter), using the Twint library, using generic keywords related to COVID-19, some of which are shown in Table 1.

**Table 1:** Keywords for Tweets.

Urdu Keywords	English Translation
کرونا	Corona
کووڈ	COVID
ویکسین	Vaccine
لاک ڈاؤن	Lockdown
این سی او سی	NCOC
آن لائن کلاسز	Online Classes
ایس او پیز	SOP’s
فیس ماسک	Face Mask
سینیٹائزر	Sanitizers
مثبت کیسز	Positive Cases
آکسیجن سلنڈر	Oxygen Cylinder
اومیکرون	Omicron
ویرئینٹ	Variant
سوشل ڈسٹنسنگ	Social Distancing
سائینوفارم	Sinopharm
سائینویک	Sinovac
اینٹی بوڈیز	Antibodies
قرنطینہ	Quarantine

Comprehensive text preprocessing was essential to effectively prepare the dataset for analysis. Our preprocessing pipeline involved several steps. First, all mentions (e.g., @username) and hashtags (e.g., #topic) were removed to eliminate non-relevant text and focus on the main content. Next, we reduced multiple spaces to a single space and removed all non-alphanumeric characters, except necessary punctuation, to standardize the text. Emoticons were extracted and replaced with corresponding sentiment labels (e.g., happy, sad), while URLs were completely removed to prevent any external content bias. Additionally, duplicated tweets were also checked and removed from the dataset. We utilized tools such as UrduHack (https://urduhack.akkefa.com/en/stable/reference/preprocessing.html) and a custom Urdu Text Preprocessing script (https://github.com/MD-Ryhan/Urdu-Text-Preprocessing) to handle language-specific challenges such as normalization of Urdu text and cleaning of various textual anomalies. The final dataset used in this study comprises 6,000 tweets. Since, for the Urdu language, a syntactic rule-based (POS) approach is used to perform ABSA, the POS tags were assigned to the tweets using publicly available CLE (https://www.cle.org.pk/index.htm) tagger that comprises a total of twelve main categories with subdivisions, resulting in 32 tags. This tagset is evaluated on 100k words of CLE Urdu digest corpus resulting in 96.8% accuracy [41].

Since there is no dataset and POS tagger available publicly for ABSA in Roman Urdu, we generate a dataset by translating 6,000 Urdu language tweets, collected from the (https://www.ijunoon.com/) website, through a crawler developed using the selenium library that automatically translates the tweets from Urdu to Roman Urdu. During the translation phase, several issues were encountered due to the linguistic complexities leading to some words not being translated into Roman Urdu. Therefore, the translated dataset is manually verified to correct the spellings of these words to ensure the quality of the dataset. These issues are as follows:

Words with “ء”: Words containing ء were not recognized and translated, e.g., “جانیۓ” (know) which contains “ء” once and “مائیکرولاک ڈاؤن” (micro lockdown) which contains “ء” two times.

Misspelled Words: Correct spellings are often ignored when using social media and many spelling mistakes were identified in the Urdu dataset that the translator could not identify and translate, e.g., the word “کلینیکل” (clinical) was written as “کلینکل” (clincl), “انکار, (reject) was written as “انقار” and “قصائی” (butcher) was written as “کسائی”.

Missing Space: In some sentences, spaces were missing between two words, hence these words were not identified and translated by the translator, e.g., in the sentence “ہزارافرادمتاثرہوۓ” (thousand people got effected), a space is missing between the words “افراد” (people) and “متاثر” (effected). Similarly, “میں مسلسل” (I consistently) was written as “میمسلسل” due to missing space.

English Loan Words: Some words do not have an English translation and are used as such with Urdu characters, e.g., “ایسٹرازینیکا” (AstraZeneca), “ٹروڈو” (Trudo), “لوزکر” (luzker), and “گائیڈلائینز” (guidelines) etc. The translator did not translate these words.

Words with Diacritics: Words containing diacritics were not properly identified by the translator, e.g., “قلؔت” (scarcity), “اللہ” (Allah), and “إنَّا ِلِلَّٰهِ وَإِنَّا إِلَيْهِ رَاجِعُونَ” (we belong to Allah and to Him we shall return).

Abbreviations: Words containing abbreviations were not identified by the translator, e.g., “سی یوآئی” (ICU) and “این سی اوسی” (NCOC).

Words Separated by Underscore: Words separated by an underscore instead of a space were also not identified by the translator, e.g., “مسجدحرام” (Holly Mosque).

Compound Words: Some compound words were not identified by the translator, e.g., “جوش و امنگ” (enthusiasm).

Words with Independent Existence: In the Urdu language, two different independent words often combine to form a single word, e.g., “دوچار” (afflicted) is a word as well as “دو” (two) and “چار” (four). These types of words were not recognized by the translator.

Consecutive Character Usage: Words containing characters appearing consecutively were also difficult for the translator to identify, e.g., in the word ”کووڈ” (COVID) and “نوول” (novel), the character “و” is appearing twice consecutively.

Other Issues: Some words were translated in Roman Urdu but with incorrect spellings, e.g., ”کرونا” (corona) was translated as “Krwana”, “ایس او پیز” (SOPs) was translated as “S O Pays”, and “مثبت” (positive) was translated as “misbet”.

### Urdu sentiment lexicon

We classify the tweets into positive, negative, or neutral sentiments using Urdu sentiment lexicon [42] that is developed by translating different lexical and linguistic resources such as SentiWordNet, English opinion word list, modifiers, English-to-Urdu-bilingual dictionary, and novel scoring mechanism. The lexicon development process, proposed by Asghar et al. [42] comprises four modules, (1) collection of English opinion words, (2) translation of opinion words in Urdu, (3) assigning scores to sentiments based on SentiWordNet and manual scoring method, and (4) collection and storage of modifiers. The resulting sentiment lexicon contains 3,651 positive sentiments, 5,730 negative sentiment, and no neutral sentiments words.

### Rule-based approach for Urdu language

In this section, we outline our methodology for extracting aspect terms, aspect term polarity, and aspect categories using syntactic rules developed specifically for Urdu. This approach involves a series of eight predefined rules that leverage the morphological and syntactic properties of Urdu, a language rich in inflection and complex sentence structures. These rules systematically identify sentiment expressions and their corresponding aspects based on their linguistic context within the sentence. To enhance clarity, we provide a detailed description of each rule, including examples that demonstrate their application. The details of these rules are as follows:

Rule 1: The polarity of opinion words present in a sentence is identified using the sentiment lexicon and then the closest noun associated with the opinions is extracted as an aspect term. In the example below, the negative **opinion term** “خطرناک” (Dangerous) is associated with the **aspect term** “کورونا” (Corona) and its polarity is **negative**.

VBF—ہے NN—خطرناک PSP—لیے PSP—کے Q—سب NN—کورونا

*Corona is dangerous for everyone.*

Rule 2: The opinion term is identified in a sentence and its associated polarity is extracted from the lexicon [19]. The associated aspect term is extracted by finding the aspect comprising of left and right nouns separated by the words “کی”, “کا”, “کے”, and “کو”. In the example below, “خطرہ” (Danger) is an **opinion term** with **negative** polarity and the multiword **aspect term** associated with it is “کورونا کی بیماری” (Corona Disease).

VBF—ہے NN—خطرناک NN—بیماری PSP—کی NN—کورونا

*Corona Disease is a danger.*

Rule 3: Firstly, sentiment words are identified and then the continuous chain of noun phrases are searched which are separated by the words “کی”, “کا”, “کے”, and “کو”. In the example below, the opinion term “تیزی” (accelerate) has a negative sentiment, and the multiword **aspect term** associated with it is “کورونا وباء کے کیسز” (cases of the corona disease) and the polarity will be **negative**.

.AUXA—گئی VBF—ہو NN—شروع VBI—آنا NN—تیزی PRP—میں NN—کیسز PSP—کے NN—وباء NN—کورونا

*Cases of the Corona disease is beginning to accelerate.*

Rule 4: After identifying the opinion term, if no phrases are found separated by the words “کی”, “کا”, “کے”, and “کو”. then search to find the continuous noun phrase chain and extract them as an aspect term. In the example below, two noun phrases i.e., i.e., “محکمہ تعلیم” (Education Department) and “کورونا وائرس” (Corona Virus) are associated with negative opinion term “نقصان” (effect/loss) hence the closest aspects will be considered as the aspect terms. Therefore, the multiword aspect terms will be “محکمہ تعلیم” (Education Department) and “کورونا وائرس” (Corona Virus) and the polarity will be **negative**.

JJ—صرف NN—وائرس NN—کرونا VBF—بتائیں NN—بات CD—ایک PRP—مجھے

AUXT—ہے VBF—دیتی NN—نقصان PSP—کو NN—تعلیم NN—محکمہ

*Tell me one thing why corona virus effects only the education department?*

Rule 5: Due to the limitation of the POS tagger, some important aspects of COVID-19 do not get the right tags. For instance, the tagger assigned tags to “ایس او پیز” (SOP’s) as NN—ایس INJ—او NN—پیز. The tagger considered the term “او” as an injective (INJ) although this should be a noun, and it should be a noun phrase consisting of three tokens. To address this issue, in this rule, we manually extract this term as an aspect. Here, “مثبت” (Positive) is the opinion word, “ایس او پیز” (SOP’s) is the aspect term having a **positive** polarity.

.AUXT—ہیں VBF—ملتے NN—نتائج JJ—مثبت PSP—سے VBI—کرنے NN—عمل PSP—پر NN—پیز INJ—او NN—ایس

*Implementing SOPs yields positive results.*

Rule 6: If an opinion term is directly associated with an adjective, then consider it as a potential aspect term [19]. In the example below,“بیان” (statement) is an opinion word, and “بڑا” (major) is an adjective term which is directly associated with the opinion term, therefore, the aspect term will be “بڑا بیان” (major statement) having a **positive** polarity.

RB—متعلق PSP—سے NN—قسم JJ—نئی PSP—کی NN—وائرس NN—کورونا

.NN—بیان JJ—بڑا PSP—کا NN—صحت NN—ادارہ JJ—عالمی

*World Health Organization’s major statement on the new strain of corona virus.*

Rule 7: If a sentence contains more than one aspect, the aspect closest to the sentiment word will be considered. In other words, the closest aspect directly associated with the opinion will be extracted. In the example below, two aspect terms are associated with the opinion word “آضافہ” (increase). Therefore, the closest aspect “ہلاکتوں کی تعداد” (death rate) will be considered as a potential multiword a aspect term having a **negative** polarity.

JJ—بھر NN—ملک PSP—سے NN—وجہ PSP—کی NN—وائرس NN—کورونا

.NN—آضافہ PSP—میں NN—تعداد PSP—کی NN—ہلاکتوں PSP—میں

*The death rate has increased across the country due to corona virus.*

Rule 8: If a sentence does not have a positive or negative sentiment, the polarity of the aspect will be considered neutral. In the example below, “ایس او پیز” (SOP’s) is not associated with a positive or negative sentiment, hence the polarity of the aspect term “کورونا ایس او پیز” (Corona SOP’s) will be **neutral**.

.NN—آگئ NN—سامنے NN—رپورٹ PSP—سے NN—حوالے PSP—کے NN—پیز INJ—او NN—ایس NN—کورونا

*The report came out regarding Corona SOPs.*

Once the ATEs are extracted, they are grouped into their respective ACTs that are summarizations of the aspects and are coarse-grained as compared to ATEs. To group the aspects into their respective categories, the extracted ATEs are analyzed, and seven ACTs are defined i.e., education, general, government policies, health, public gatherings, travel, and others by manually analyzing the ATEs. The polarity of the ACTs is considered the same as ATEs. The health category deals with different aspects i.e., deaths, COVID-19 cases, recoveries, hospitals, and ventilators. Similarly, the education category comprises aspects like online classes, online exams, holidays, etc. The public gathering comprises marriage gatherings, social gatherings, etc. The government policies category contains aspects like NCOC, SOP’s, lockdown, and the travel category deals with the ban on travel and its related aspects. If a tweet contains a generic description of COVID-19, it is assigned to the general category and if a tweet discusses none of these categories, it is assigned to the others category. Tables 2 and 3 show the statistics after the proposed syntactic rules are applied to the Urdu dataset.

**Table 2:** Statistics of ATE and ATP.

Polarity	Aspects Terms	Potential Aspects	Aspect Term Polarity
Positive	6,223	1,175	1,175
Negative	6,403	3,947	3,947
Neutral	2,551	878	878
Total	15,177	6,000	6,000

**Table 3:** Statistics of ACT.

Aspect Category	Count
Education	42
General	1,106
Government Policies	248
Health	517
Other	550
Public Gatherings	27
Travel	8

### Unsupervised approach for Roman Urdu

For Roman Urdu, instead of adopting a rule-based approach, aspects are extracted from the data based on collocations because there is no POS tagger available to tag Roman Urdu data. Collocations are used to extract the ATEs from the tweets that mainly refer to the lexical relationship of two or more words that frequently occur together and make a common expression with a meaning that can be derived from at least one of its components [43]. This technique helps in the identification of phrases related to a particular aspect, even if they are not present adjacently in the text. Firstly, bigram collocations are extracted from the translated Roman Urdu dataset. A total of 2,468 collocations are extracted from the data. Then, the strength of association between the extracted collocations is extracted using point-wise mutual information (PMI) which is a good measure to find association among words based on information theory. It works by comparing the probability of two words that occur together with the probability of observing them independently. It estimates whether the two words are genuinely associated with one another or are placed together by chance. The mutual information of two words with probabilities *P*(*word*_1_) and *P*(*word*_2_) can be defined using Equation 1. The probability of individual words *P*(*word*_1_), *P*(*word*_2_) and their combined probabilities $P(word_{1},word_{2})$ can be observed by counting the number of occurrences of *word*_1_, *word*_2_ and the co-occurrence of *word*_1_ and *word*_2_ in a dataset normalized by the size of the dataset [44].

$$PMI(word_{1},word_{2})=log\left[\frac{P(word_{1}\&word_{2})}{P(word_{1}),P(word_{2})}\right]$$ (1)

Latent Dirichlet Allocation Algorithm (LDA) [45] is used to find ACT which is coarse grain as compared to ATEs. LDA is a probabilistic topic modeling approach that represents the document as an arbitrary blend over a set of random topics. Each topic is characterized by a distribution of vocabulary. LDA is categorized into two processes i.e., generative process and inference process. The generative process generates the documents in which the values of word-topic probability $\left(\phi_{k}\right)$ and the proportion of topic for each document $\left(\theta_{d}\right)$ are known. Further, the inference process finds the value of latent variables i.e., word-topic probability and topic proportion of the already known documents. The equation to find the probability of word topic and topic proportion is given below.

$$\phi_{k}=p(w=t|z=k)=\frac{n_{t,k}+\beta_{t}}{\sum_{t=1}^{V}n_{t,k}+\beta_{t}}$$ (2)

$$\theta_{d}=p(z=k|d)=\frac{n_{d,k}+\alpha_{k}}{\sum_{k=1}^{K}n_{d,k}+\alpha_{k}}$$ (3)

## Experimental setup

Experiments on Urdu tweets are performed using the scikit-learn toolkit, and three types of fine-grained information are extracted from the dataset including ATEs, ATP, and ACT using the eight rules defined previously. Accuracy, precision, recall, and F1-score are used as evaluation measures. Accuracy is the ratio of correctly classified instances to the total number of instances [46]. Precision is the ratio of the number of instances correctly classified as positive to the total number of positively classified instances [46]. Recall is the ratio of instances classified as positive to the total number of truly positive instances [46]. F1-score is the harmonic mean of precision and recall [46].

$$Accuracy(A)=\frac{T_{P}+T_{N}}{T_{P}+T_{N}+F_{P}+F_{N}}$$ (4)

$$Precision(P)=\frac{T_{P}}{T_{P}+F_{P}}$$ (5)

$$Recall(R)=\frac{T_{P}}{T_{P}+F_{N}}$$ (6)

$$F1-score(F)=\frac{2\times Precision\times Recall}{Precision+Recall}$$ (7)

ABSA is performed on Roman Urdu tweets using the sci-kit learn tool kit and two classification tasks i.e., extraction of ATEs and the extraction of ACT. The evaluation of ATEs that are extracted using collocation patterns with PMI is performed using t-test by considering the t value of 0.05 using Equation 8, where “*x*” and “μ” represent mean values, “*s*” shows standard deviation, “*z*” illustrates the standard error added or subtracted to achieve the required confidence level, and “*n*” is the sample size.

$$t=\frac{\left(x-\mu \right)}{s/\sqrt{n}}\;\;\;and\;\;\;CI=\left[x-z\left(\frac{s}{\sqrt{n}}\right),x+z\left(\frac{s}{\sqrt{n}}\right)\right]$$ (8)

The evaluation of LDA is performed using two metrics i.e., coherence and perplexity. Coherence measures the meaningfulness and interpretability of topics generated by LDA. It also measures the semantic similarity among the words that are present within the topics. The greater value of coherence shows more meaningful and coherent topics [47]. Perplexity computes how well the model predicts unseen data. A lower value of perplexity shows the better performance of the LDA model [45]. Equations 9 and 10 compute coherence [48] and perplexity [47].

$$Coherence=\frac{1}{C}*\sum PMI(w_{i},w_{j})$$ (9)

$$Perplexity=exp\left[-1*\frac{\text{log likelihood}}{\text{total number of words}}\right]$$ (10)

Hyperparameter tuning of LDA is performed by performing several experiments and the best parameters obtained, that we used are shown in Table 4.

**Table 4:** Hyperparameter tuning of LDA model.

Parameters	LDA Model
No. of Topics	7
Random State	100
Update Every	1
Chunk Size	100
Passes	10
Alpha	Auto
Pre_Word_Topics	True

## Results and discussion

### Rule-based approach for Urdu language

The efficiency of the proposed approach is checked by manually annotating the instances of COVID-19 tweets. We manually extract ATEs, ATP, ACT, and ACP from the dataset. Then, ATEs, ATP, ACT, and ACP are extracted from the dataset using the proposed rule-based approach. For the extraction of ATP, the Urdu sentiment lexicon is used comprising 3,651 positive and 5,730 negative sentiment words. The performance of manual annotation of ATEs, ATP, ACT, and ACP is compared with those predicted by the proposed rule-based approach as shown in Table 5.

**Table 5:** Results of rule-based approach for Urdu language.

Classification Task	Accuracy	Precision	Recall	F1-score
Aspect Term/s	83%	79%	83%	80%
Aspect Term/s Polarity	74%	83%	74%	75%
Aspect Category	71%	73%	71%	70%
Aspect Category Polarity	74%	83%	84%	75%

Although the achieved F1-scores are satisfactory, we encountered several issues that if addressed can further improve the performance of the proposed approach. For instance, the performance of the syntactic rule-based approach is highly dependent on the POS tagger. However, due to the nature of our dataset, some words could not get quality POS tagging. In the example below, the extracted aspect term should be “شہریار آفریدی کا کرونا” (Corona test of Shehryar Afridi) against the opinion term “منفی” (negative) but it is extracted as “بعد شہریار آفریدی کا کرونا”.

NN—شہریار NN—بعد PSP—کے VBI—رہنے PSP—میں NN—قرنطینہ

NN—آگیا JJ—منفی NN—کرونا PSP—کا NN—آفریدی

*After living in quarantine, the Corona test of Shahriar Afridi turned out negative.*

Numbers are very important in our dataset as they portray the death rates and recoveries of the patients. These numbers are not removed from the data during the preprocessing phase. Some of the information about the aspects is incomplete because they are not incorporated while defining the rules. In the example below, the opinion word is “مثبت” (positive) and the extracted aspect is “فیصد کیسز”(% cases) which is not conveying the quality of information about the aspect.

NN—شدہ NN—رپورٹ PSP—کے NN—کورونا PSP—میں NNP—اٹلی

VBF—ہیں JJ—مثبت NN—کیسز NN—فیصد PSP—96

*In Italy 96% of reported cases of corona are positive.*

Additionally, our main perception was that the syntactic rule-based approach is domain-independent as it extracts the aspects and their polarities based on syntactic patterns however, in the above example, it is observed that the opinion word “مثبت” (positive) shows the positive opinion. However, after analyzing the context of the sentence it is negative as the patients of COVID-19 are up to 96% in Italy. Depending on the context its polarity should be negative, however, it is assigned as positive by the proposed rule-based approach.

Moreover, it is also observed that the efficiency of the rule-based method is dependent on the coverage of the lexicon and the accuracy of the Urdu text written by the user. Many X (formerly Twitter) users do not follow the language standard while writing on OSNs. Consequently, misspelled words are not correctly identified by the tagger resulting in the wrong assignment of the POS tag and affecting the performance of the aspect extraction process. Similarly, lexicons are mostly built in a standard format of the language, hence the misspelled words usually remain uncategorized. To address this, we found that by applying spell-checking methods before aspect extraction and aspect polarity identification further enhances the efficiency of the rule-based approach.

### Comparison of rule-based approach with state-of-the-art

We compare our work with the existing state-of-the-art method on Urdu ABSA. According to the standard provided by SemEval [49], there are four classification tasks to perform ABSA: extraction of Aspect Term/s (ATEs), Aspect Term Polarity (ATP), Aspect Category (ACT), and Aspect Category Polarity (ACP). However, the existing study [19] focuses solely on one task, ATEs. In contrast, our study addresses three of these tasks—ATEs, ATP, and ACT—treating ACP equivalently to ATP. For comparison purposes, we conduct experiments within the context of COVID-19 as well as on the datasets covering a broader range of topics. Our approach outperforms the existing state-of-the-art study by an improved F1-score of 5% for ATEs. This enhanced performance is primarily due to the six syntactic rules we developed for ATE extraction, compared to the two rules employed in the previous study. These results are documented in Table 6 and underscore the adaptability of our methods to diverse sentiment analysis scenarios, demonstrating their potential applicability beyond pandemic-related discourse.

**Table 6:** Comparison with state-of-the-art-method (P = Precision, R = Recall, F = F1-score).

Ref	Language	Technique	ATE	ATP	ACT	P	R	F
[19]	Urdu	Syntactic Rule Based	**✓**	**✗**	**✗**	78%	76%	76%
Proposed Work	Urdu	Syntactic Rule Based	**✓**	**✓**	**✓**	81%	81%	81%

### Statistical T-test for significant aspects in Roman Urdu

Aspect terms are identified from the tweets using the collocation technique. For this purpose, stop words are removed from the tweets to avoid filtering them as a significant aspect and to reduce noise from the data. For this, the bigram approach is used and initially, 51,149 collocations are identified from the data. To filter out the significant aspects from the extracted collocation, PMI is used which shows the strength of the association between the two words. The PMI values of extracted collocations from our dataset range from [−15,15]. According to the PMI, it is assumed that the words that are closer to zero are significantly associated with each other and they do not occur by chance. Therefore, lower PMI values show more association between two words and vice versa. To evaluate this and to determine the best PMI value for our dataset, a statistical t-test is used on the extracted collocations. For this purpose, a total of 7 experiments are performed and each experiment contains two samples i.e., significant aspects and non-significant aspects. The details of these experiments are listed in Table 7. To check whether these samples are statistically significant or not, our Null Hypothesis (*H*_0_) and Alternative Hypothesis (*H*_*A*_) are as follows:

*H*_0_: The selected collocations are not significant.

*H*_*A*_: The selected collocations are significant.

**Table 7:** Ranges of significant and not-significant ATEs for Roman Urdu.

Experiment	Significant Aspects Range	Non-significant Aspects Range
1.	[−1,1]	[>±1 and ≤±15]
2.	[−2,2]	[>±2 and ≤±15]
3.	[−3,3]	[>±3 and ≤±15]
4.	[−4,4]	[>±4 and ≤±15]
5.	[−5,5]	[>±5 and ≤±15]
6.	[−6,6]	[>±6 and ≤±15]
7.	[−7,7]	[>±7 and ≤±15]

The significance of the hypothesis is statistically evaluated using a two-tailed t-test (t) and confidence interval (CI) with a significance level of 0.05 on the samples of the aspects. Our null hypothesis is accepted for experiments 2 to 7. For expriment 1, our alternative hypothesis is accepted. Therefore, by considering this experiment we chose the collocations with values ranging from [−1,1] as significant aspect terms in our dataset, and a total of 2,648 collocations are identified as significant aspect terms in our dataset.

### LDA for aspect categories in Roman Urdu

To identify the aspect categories, the LDA model is trained on the Roman Urdu dataset comprising COVID-19 related tweets. The performance of LDA is checked by considering two evaluation measures i.e., perplexity and coherence. Perplexity quantifies how well a model generalizes the unseen data. A lower value of perplexity indicates better performance of the model. In this research, we achieve the perplexity value of -7, as shown in Table 8, indicating the strong capability of the model to predict the unseen aspects in the given dataset. It also shows that our model effectively finds the aspect categories for SA and accurately captures the relation among the words and aspect categories within the dataset, thus empowering it to predict unseen aspect categories with high precision.

**Table 8:** Results of LDA model for ACT on Roman Urdu dataset.

Evaluation Measures	Score
Perplexity	-7
Coherence	41

Coherence quantifies how well the topics are semantically related to each other. We assess the coherence of identified aspect categories and our model achieves the coherence value of 41, as shown in Table 8. This indicates that the extracted categories are semantically related and offer a coherent illustration of underlying sentiment-bearing topics in a dataset. It highlights the quality of aspect categories identified by LDA. The high value of coherence indicates that the generated aspect categories capture significant and semantically coherent patterns in the dataset. Additionally, it shows that this model successfully grouped the words that are closely associated in terms of sentiments, assisting improved understanding of aspects in reviews. Additionally, the combination of high coherence and low perplexity score shows the effectiveness and strong performance of the LDA model for the identification of aspect categories demonstrating the ability of the model to represent and uncover the latent sentiment-bearing aspect categories in the dataset which is important for the comprehension of overall sentiment expressed in the dataset. The limitation of this study is that the evaluation of the model is performed on a domain-specific dataset i.e., COVID-19 tweets, therefore it might be possible that the results may vary when this model is applied to other domains. Moreover, the choice of hyperparameters and preprocessing techniques may impact the interpretability and performance of the LDA model.

## Conclusion

This study presents BI-SENT, an unsupervised approach for bilingual aspect-based sentiment analysis of COVID-19 tweets for the Urdu language and Roman Urdu. Due to the difference in writing styles of both languages, we handle them separately rather than considering the text as code-mixed. Due to this, a parallel corpus is developed on COVID-19 for both languages. For the Urdu language, a syntactic rule-based approach is used to extract the ATEs, ATPs, and ACTs. For this purpose, we crafted six syntactic rules and achieved a 5% improvement in F1-score as compared to state-of-the-art. Additionally, it is also effective in the extraction of ATPs, and ACT. For Roman Urdu, collocations are used to extract ATEs, and topic modeling is used to extract ACTs. Our experimental results show the extracted categories are semantically related and offer a coherent illustration of underlying sentiment-bearing topics in a dataset. It highlights the quality of ACTs identified by LDA. The high coherence value indicates that the generated aspect categories capture significant and semantically coherent patterns in the dataset. During this research, we found that the accuracy of the unsupervised approach is domain-dependent and does not capture the contextual information in polarity identification. It is also dependent on the accuracy of the POS tagger, and the coverage of the lexicon. Moreover, the use of non-standard words by X (formerly Twitter) users also affects the accuracy of the proposed methods as lexicons are developed using standard word formats. In addition to this, it is also observed that the most discussed topics during the pandemic were health, education, public gatherings, government policies, and travel in Pakistan. In the future, we plan to use a spell checker during the preprocessing process to enhance the accuracy of the proposed method. Additionally, considering the potential benefits of contextualized word embeddings for understanding nuanced expressions, we aim to explore hybrid approaches in future studies. These would potentially integrate advanced NLP techniques, such as BERT, with our existing rule-based system. This integration aims to better capture the context and subtleties of sentiment expressed in non-standard language forms, enhancing our model’s robustness and applicability to a broader range of textual data. Additionally, we plan to develop a POS tagger and robust normalization techniques for Roman Urdu to standardize text processing and improve sentiment analysis performance. Our research will further investigate a hybrid approach that combines the linguistic precision of rule-based methods with the robustness of machine learning models, aiming to address the limitations of collocation-based methods and enhance analytic efficacy in Roman Urdu aspect-based sentiment analysis.
